# 155. Long-Acting Subcutaneous Lenacapavir in People with Multi-Drug Resistant HIV-1: 3-Year Results of the CAPELLA Study

**DOI:** 10.1093/ofid/ofae631.041

**Published:** 2025-01-29

**Authors:** Onyema Ogbuagu, Joseph P McGowan, Ann Stapleton, Andrew Wiznia, Daniel Berger, Catherine M Creticos, Debbie P Hagins, Olayemi Osiyemi, James Sims, David A Wheeler, Hui Wang, Nicolas A Margot, Hadas Dvory-Sobol, Martin Rhee, Sorana Segal-Maurer

**Affiliations:** Yale School of Medicine, Cheshire, CT; Center for AIDS Research and Treatment, Manhasset, New York; Rimrock Clinic, Eisenhower Health, Rancho Mirage, California; Jacobi Medical Center, Albert Einstein College of Medicine, Bronx, New York; Northstar Medical Center, Chicago, Illinois; Howard Brown Health, Chicago, Illinois; Georgia Department of Public Health, Coastal Health District, Chatham CARE Center, Savannah, GA, USA, Savannah, Georgia; Triple O Research Institute, West Palm Beach, Florida; St Hope Foundation, Houston, Texas; Infectious Diseases Physicians, Inc., Annandale, Virginia; Gilead Sciences Inc., Foster City, California; Gilead Sciences Inc., Foster City, California; Gilead Sciences, Foster City, California; Gilead Sciences, Foster City, California; Division of Infectious Diseases, New York–Presbyterian Queens, Flushing, New York

## Abstract

**Background:**

Lenacapavir (LEN) is a highly potent, long-acting HIV-1 capsid inhibitor approved in combination with other antiretrovirals (ARVs), for the treatment of heavily treatment-experienced (HTE) people with HIV-1 (PWH) with multidrug resistance (MDR), based on the Phase 2/3 CAPELLA study. Data from CAPELLA were previously reported for Week (W) 26, W52, and W104. At W104, most participants had virologic suppression, including those with no fully active ARVs. Here, we report W156 efficacy and safety results.

**Table:**
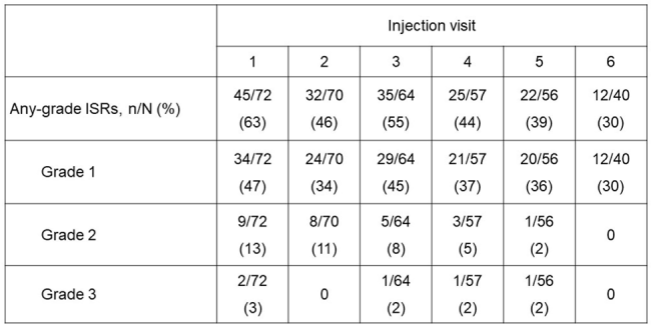
Injection Site Reactions Following Each Injection

ISR, injection site reaction.

**Methods:**

In CAPELLA (NCT04150068), participants received LEN oral initiation followed by subcutaneous (SC) LEN every 6 months combined with an OBR. Endpoints included virologic outcomes, CD4 cell count, adverse events, and LEN resistance.

**Results:**

We enrolled 72 participants: 25% female; 38% Black; median age, 52 years; CD4 cell count < 200 cells/μL, 64% (< 50 cells/μL, 22%). At W156, 61% (43/70) had HIV-1 RNA < 50 copies/mL by FDA Snapshot Algorithm, 16% (11/70) had ≥ 50 copies/mL, and 23% (16/70) had no data in window (of n=16 without data: discontinued before W156, n=14; suppressed at next visit, n=1; > 50 copies/mL at next visit, n=1). By missing=excluded analysis, 85% (44/52) had HIV-1 RNA < 50 copies/mL. CD4 cell count increased throughout the study; median (IQR) increase from baseline to W156 was 115 (59–233) cells/μL. At W156, 22% had CD4 < 200 cells/μL. No new cases of LEN resistance emerged after the W104 analysis. Two participants discontinued study drug due to Grade 1 injection site nodules (n=1 previously reported). Injection site reactions (ISRs) were mostly Grade 1/2, and frequency declined with later injections (**Table**). Overall, 98% of participants received SC injections within ± 14 days of their scheduled visits.

**Conclusion:**

LEN plus an OBR maintained a high rate of virologic suppression over 3 years. The safety profile of LEN was consistent with prior analyses. Adherence to LEN injections was high, possibly due to participant motivation and favorable LEN tolerability. These longer-term data demonstrate continued efficacy and safety of LEN for HTE PWH with MDR.

**Disclosures:**

**Onyema Ogbuagu, MD**, Gilead Sciences, Inc.: Advisor/Consultant|Gilead Sciences, Inc.: Honoraria|GSK/ViiV: Advisor/Consultant|GSK/ViiV: Honoraria|Janssen: Advisor/Consultant|Moderna: Advisor/Consultant|Moderna: Honoraria **Ann Stapleton, MD**, GSK: Advisor/Consultant **Andrew Wiznia, MD**, Gilead Sciences, Inc.: Advisor/Consultant|Janssen: Advisor/Consultant **Daniel Berger, MD**, Gilead Sciences: Stocks/Bonds (Private Company) **Catherine M. Creticos, MD**, Gilead Sciences, Inc.: Speaker|Theratechnologies: Speaker|ViiV: Speaker **Debbie P. Hagins, MD, FAPCR, AAHIVS**, Gilead Sciences, Inc.: Medical writing support provided by Aspire Scientific (Bollington, UK) **Olayemi Osiyemi, MD**, Gilead Sciences, Inc.: Advisor/Consultant|Gilead Sciences, Inc.: Speaker bureau|Merck: Advisor/Consultant|ViiV: Advisor/Consultant|ViiV: Speaker bureau **David A. Wheeler, MD**, AstraZeneca: Grant/Research Support|Gilead: Grant/Research Support|Janssen: Grant/Research Support **Hui Wang, PhD**, Gilead Sciences, Inc.: Employee|Gilead Sciences, Inc.: Stocks/Bonds (Public Company) **Nicolas A. Margot, MA**, Gilead Sciences, Inc.: Employee|Gilead Sciences, Inc.: Stocks/Bonds (Public Company) **Hadas Dvory-Sobol, PhD**, Gilead Sciences, Inc.: employee and shareholder **Martin Rhee, MD**, Gilead Sciences, Inc.: employee and shareholder **Sorana Segal-Maurer, MD**, Gilead Sciences, Inc.: Advisor/Consultant|Gilead Sciences, Inc.: Grant/Research Support|Gilead Sciences, Inc.: Honoraria|Janssen: Advisor/Consultant|Theratechnologies: Advisor/Consultant|ViiV: Advisor/Consultant

